# *Bcl10* mutations in malignancy

**DOI:** 10.1038/sj.bjc.6690549

**Published:** 1999-07

**Authors:** M J S Dyer

**Affiliations:** Academic Haematology and Cytogenetics, Haddow Laboratories, Sutton, Surrey, SM2 5NG, UK


*British Journal of Cancer* (1999) **80**(10), 1491
© 1999 Cancer Research Campaign
Article no. bjoc.1999.0549

Editorial

### *Bcl10* mutations in malignancy

**MJS Dyer**

Academic Haematology and Cytogenetics, Haddow Laboratories, Institute of Cancer Research, Sutton, Surrey SM2 5NG, UK

This issue of the *British Journal of Cancer* contains four reports showing a lack of mutation of *Bcl10* in genomic DNA from malignancies of different histological subtypes. These data appear to be at variance with our original report implicating *Bcl10* mutations in the pathogenesis of a wide range of turnours (Willis et al, 1999). However, from our further work it now seems that at least some of the *Bcl10* abnormalities, originally described as mutations, in fact represented post-transcriptional RNA modification of *Bcl10* and are not encoded in genomic DNA.

*Bcl10* was cloned through its direct involvement in chromosomal translocation t(1;14)(p22;q32), a rare but recurrent translocation of MALT lymphoma (Willis et al, 1999). The presence of an amino-terminal caspase recruitment domain in *Bcl10* suggested a role in the apoptotic cascade, and from its involvement in the chromosomal translocation it was anticipated that *Bcl10*, like *Bcl2*, would be anti-apoptotic. However, wild-type *Bcl10* is pro-apoptotic and has the features of a tumour suppressor gene (Koseki et al, 1999; Thome et al, 1999; Willis et al, 1999; Yan et al, 1999; Zhang et al, 1999).

We therefore sought *Bcl10* mutations first in cDNA clones from one MALT lymphoma with t(1;14)(p22:q32). These comprised point mutations, which were often multiple, deletions at the exon 3–4 boundary, and insertions and deletions within two homopolymeric runs of eight adenines and seven thymidines. An identical spectrum of cDNA abnormalities has been detected independently in other cases of t(1;14)(p22;q32) MALT lymphoma (Zhang et al, 1999), making it unlikely that all these changes represented polymerase chain reaction or cloning artefacts. Thus, in MALT lymphoma with t(1;14)(p22;q32) there were multiple and ongoing *Bcl10* abnormalities. We assumed initially that these reflected the indolent behaviour of MALT lymphoma and that with further in vivo selection, a dominant clone would ultimately emerge.

However, we have now sequenced multiple cDNA clones from various malignant cell lines, primary lymphoid tumours and normal tissues and found all three types of abnormality at varying frequency in all instances. The data presented originally (Table 2 in Willis et al, 1999) referred to cDNA clones from six malignant cell lines, including Tera-1, Tera-2, GCT-44 and from three mesothelioma lines, and in all, multiple *Bcl10* abnormalities were detected. In contrast, a much lower mutational frequency was detected in genomic DNA. Also, multiple cDNA abnormalities have been detected in some cell lines, including the Tera-2 cell line in the presence of apparently normal germline DNA (Dyer et al, 1999).

To summarize the three types of deviation from the wild-type sequence:

a. *Bcl10* utilizes three alternative splice sites at the exon 3–4 boundary
b. Alterations within the homopolymeric runs of eight As and seven Ts are common in *Bcl10* cDNAs, and in most instances are not templated
c. *Bcl10* point mutations are multiple and ongoing. Whether all point mutations are present in genomic DNA or whether some are non-templated is not clear.

*Bcl10* therefore appears to undergo what has been termed ‘molecular misreading’ (van Leeuwen et al, 1998) and at least some of the discrepancies between our initial data and those reported here can be ascribed to our use of cDNA rather than genomic DNA.

### REFERENCES

Dyer MJS, Price H, Jadayel DM, Gasco M, Perry AR, Hamoudi RA, Willis TG, Peng H, Du M-Q and Isaacson PG (1999) *Bcl10* abnormalities in malignancy. *Cell* (in press)

Koseki T, Inohara N, Chen S, Carrio R, Merino J, Hottiger MO, Nabel GJ and Nunez G (1999) CIPER, a novel NF-κB-activating protein containing a caspase recruitment domain with homology to *Herpesvirus*-2 Protein E10. *J Biol Chem* **274**: 9955–9961

van Leeuwen FW, Burbach JPH and Hol EM (1998) Mutations in RNA: a first example of molecular misreading in Alzheimer’s disease. *Trends Neurosci* **21**: 331–335

Thome M, Martinon F, Hofmann K, Rubio V, Steiner V, Schneider P, Mattmann C and Tschopp J (1999) Equine *Herpesvirus*-2 E10 gene product, but not its cellular homologue, activates NF-κB transcription factor and c-Jun N-terminal kinase. *J Biol Chem* **274**: 9962–9968

Willis TG. Jadayel DM, Du M-Q, Peng H, Perry AR, Abdul-Rauf M, Price, H, Karran L, Majekodunml O, Wlodarska I, Pan L, Crook T, Hamoudi RA, Isaacson PG and Dyer MJS (1999) *Bcl10* is involved in t(1;14)(p22;q32) of MALT B cell lymphoma and mutated in multiple tumor types. *Cell* **96**: 35–45

Yan M, Lee J, Schilbach S, Goddard A and Dixit VM (1999) mE10, a novel caspase recruitment domain-containing pro-apoptotic molecule. *J Biol Chem* **274**: 10287–10292

Zhang Q, Siebert R, Yan M, Hinzmann B, Cui X, Xue L, Rakestraw KM, Naeve CW, Beckmann G, Weisenberger DD, Sanger WG, Nowotny H, Vesely M, Callet-Bauchu E, Salles G, Dixit VM, Rosenthal A, Schlegelberger B and Morris SW (1999) Inactivating mutations and overexpression of *Bcl10*, a caspase recruitment domain (CARD)-containing gene in MALT lymphoma with t(1;14)(p22;q32). *Nat Genet* **22**: 63–68

*Received 4 May 1999*
*Accepted 4 May 1999*

*Correspondence to:* MJS Dyer

